# Yinchenhao Tang alleviates high fat diet induced NAFLD by increasing NR1H4 and APOA1 expression

**DOI:** 10.1016/j.jtcme.2023.02.010

**Published:** 2023-02-24

**Authors:** Li Xu, Hongliang Cui

**Affiliations:** Medical Experiment Center, Shaanxi University of Chinese Medicine, Xianyang, 712046, China

**Keywords:** NAFLD, Yinchenhao tang, Lipid metabolism, NR1H4, APOA1

## Abstract

**Background and aim:**

Traditional Chinese medicine Yinchenhao Tang (YCHT) demonstrated benefits when treating nonalcoholic fatty liver disease (NAFLD), but the dose effects and potential targets are still ambiguous. In this study, different concentrations of YCHT were employed to treat NAFLD and the underlying therapeutic targets were investigated.

**Experimental procedure:**

Kunming mice were fed with high fat diet (HFD) for 8 weeks to induce NAFLD, then treated with 3 different concentrations of YCHT. Hepatic pathological changes and serum lipid levels were examined. Network pharmacology was applied to screen the potential targets of YCHT for NAFLD modulation. NR1H4 and APOA1 expression was evaluated by QPCR and western blotting. Immunohistochemistry (IHC) staining was conducted to visualize the localization pattern of NR1H4 and APOA1 in the liver.

**Results:**

YCHT significantly reduced liver lipid storage and improved the liver pathological status of NAFLD mice. The serum lipid levels, as well as alanine aminotransferase (ALT) and aspartate aminotransferase (AST) levels, were remarkably reduced by the middle and high dose YCHT. There are 35 potential targets for YCHT to regulate NAFLD. HFD suppressed both RNA and protein expression of NR1H4 and APOA1, while YCHT elevated NR1H4 and APOA1 expression. IHC staining indicated that NR1H4 was mainly located in the cell nucleus and the APOA1 signal was observed at the liver sinusoid or cytoplasm.

**Conclusion:**

YCHT can effectively ameliorate HFD induced NAFLD by modulating the promising targets of NR1H4 and APOA1.

## Highlights of the findings and novelties

• YCHT in middle or high dose has better effects when treating high fat diet induced NAFLD.
• YCHT upregulated NR1H4 and APOA1 expression in RNA and protein levels which were suppressed by NAFLD.
• YCHT can improve high fat diet induced NAFLD by increasing NR1H4 and APOA1 expression.

## Graphical abstract

**Figure:**
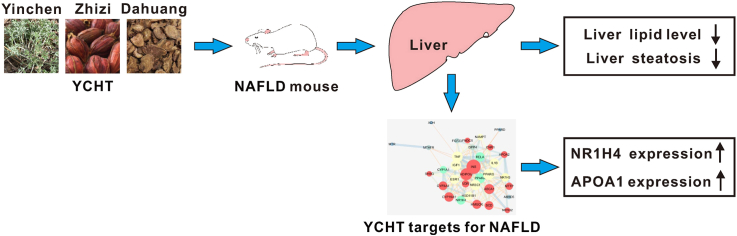

## List of abbreviations

ADIPOQ: adiponectin
PPARG: peroxisome proliferator activated receptor gamma
IGF1: insulin-like growth factor 1
PPARA: peroxisome proliferator activated receptor alpha
MTHFR: methylenetetrahydrofolate reductase
SERPINE1: serpin family E member 1
NR1H4: nuclear receptor subfamily 1 group H member 4
APOA1: apolipoprotein A1
APOA2: apolipoprotein A2
FBG: fast blood glucose
TG: triglyceride
TCHO: total cholesterol
LDL-C: low density lipoprotein cholesterol
HDL-C: high density lipoprotein cholesterol
ALT: alanine aminotransferase
AST: aspartate aminotransferase

## 1 Introduction

Nonalcoholic fatty liver disease (NAFLD) is nowadays a prevalent chronic disease that parallels the expanding global obesity population.^1^ NAFLD occurrence is closely associated with obesity and diabetes^2^ which are normally caused by high calorie food intake and absence of exercise. NAFLD usually manifests as nonalcoholic fatty liver (NAFL) or nonalcoholic steatohepatitis (NASH). As the development of NAFLD, it can progress to cirrhosis, hepatocellular carcinoma, and even death.^3^ However, lacking approved drugs or treatments^4^ makes NAFLD a huge public health burden.

It has been reported that many traditional Chinese herbs including the famous Chinese medicine formula Yinchenhao Tang (YCHT) have noticeable therapeutic effects in the treatment of NAFLD.^5^ YCHT contains *Artemisia capillaris* Thunb., *Gardenia jasminoides* J. Ellis, and *Rheum Palmatum* L. which was first recorded by “*Shanghanlun*” in 200–210 A.D. Han Dynasty of China. YCHT was widely prescribed in Asian countries to treat jaundice and liver diseases,^6^ recently more and more studies support using it as an efficient treatment for NAFLD.^7^^,^^8^

YCHT can inhibit hepatic free fatty acid (FFA) accumulation and alleviate liver steatosis of high fat diet (HFD) induced NAFLD mice.^9^ Moreover, YCHT can also improve liver ballooning degeneration and reduce serum alanine aminotransferase (ALT) levels in HFD induced NASH mice.^10^ For NASH rats, YCHT demonstrated the ability to decrease not only hepatic triglyceride (TG) and FFA contents, but also the serum tumor necrosis factor-α (TNF-α) levels.^11^ As the progress of NAFLD, it has a high chance to deteriorate into liver cirrhosis.^12^ In addition, YCHT has also been reported to down-regulate liver fibrosis related protein level^13^ and suppresses liver fibrosis by modulating apoptosis related signaling pathways.^14^

Even though YCHT exhibits great potential to prevent NAFLD and its induced hepatic diseases. However, different results were reported during YCHT usage in NAFLD management, and the mechanism of YCHT for NAFLD modulation has not been fully illustrated. In this study, 3 different concentrations of YCHT were applied to treat HFD induced NAFLD mice. Network pharmacology was utilized to screen the potential targets and pathways. Finally, the predicted targets for YCHT to modulate NAFLD were further verified in RNA and protein levels.

## 2 Material and methods

### 2.1 Preparation of YCHT

All the herbs were provided by Shaanxi University of Chinese Medicine school hospital. *Artemisia capillaris* Thunb. (Yinchen; Shannxi; Lot No.: 201,701,109), *Gardenia jasminoides* J. Ellis (Zhizi; Jiangxi; Lot No.: 170101) and *Rheum Palmatum* L. (Dahuang; Gansu; Lot No.: 20170501) with a ratio of 18:9:6 were extracted as the following procedure to make YCHT decoction. 90 g Artemisia capillaris Thunb. Was firstly soaked in 1650 ml water for 1 h, and then boiled for 30 min. Secondly, 45 g ground *Gardenia jasminoides* J. Ellis and 30 g *Rheum Palmatum* L. were added to the decoction to boil for another 15 min, after which the decoction was collected. Another 1320 ml of fresh water was added to the formula and boiled for 20 min, then the second batch of the decoction was gathered. Finally, the concentrated YCHT decoction mixture from both batches was prepared by a rotary evaporator at 60 °C and stored at 4 °C for further use. Gallic acid, chlorogenic acid, geniposide, and crocin have been declared as the main index compounds in the aqueous extract of YCHT.^15^

### 2.2 Animals and experimental procedure

Male Kunming mice (body weight ranged from 30 to 32 g) were purchased from the Experimental Animal Center of Xi'an Jiaotong University (SCXK 2012–003). Animals were housed at 25 °C with a 12 h/12 h light-dark cycle and free access to food and water. All animal experiments were carried out in accordance with the National Institutes of Health guide for the care and use of laboratory animals. All animal studies were approved by the Animal Experiment Committee of Shaanxi University of Chinese Medicine (SUCMDL20230113002).

After one week of acclimatization, 10 mice fed with standard chow were randomly selected as the control group (Control). 32 mice fed with high fat diet (HFD) were evenly divided as the NAFLD group (NAFLD), YCHT low dose group (YCHT L), YCHT middle dose group (YCHT M), and YCHT high dose group (YCHT H). HFD comprises 10% lard, 5% yolk, 5% sucrose, 2% cholesterol, 0.5% Cholate, and 77.5% standard chow.

After 8 weeks, mice from all the groups were intragastrically administered with saline or YCHT decoction once a day for 4 weeks. Control mice were fed with standard chow during the treatment and administered saline. NAFLD mice were fed HFD and administered saline. YCHT L mice were fed HFD and administered with YCHT decoction equal to 2.06 g/kg crude herbs. YCHT M mice were fed HFD and administered with YCHT equal to 4.125 g/kg crude herbs. YCHT H mice were fed HFD and administered decoction equal to 6.19 g/kg crude herbs.

Animals fasted for 12 h before sample collection. Mice in each group were anesthetized with isoflurane, after which blood and liver tissues were collected. Fasting blood glucose (FBG) levels and liver weight was measured during the sample collection.

### 2.3 Histopathology assay

Oil red O staining: Fresh liver tissues were fixed with 4% paraformaldehyde and then prepared as 10 μm thick frozen sections. Air-dried frozen sections were soaked in distilled water for 10 min and then briefly rinsed with 60% isopropanol. After being stained with freshly prepared oil red O working solution for 10 min, sections were rinsed with 60% isopropanol. Following by distilled water washing, sections were stained with Mayer's hematoxylin for 10 s. Afterward, the stained sections were thoroughly washed in running water for 5 min. Finally, before image acquisition, the sections were rinsed with distilled water and mounted with glycerin.

Periodic acid Schiff (PAS) staining: The wax embedded liver tissues were prepared as 3 μm thick paraffin sections for glycogen detection. Liver glycogen was detected using a Periodic Acid Schiff Stain Kit (G1281, Solarbio, Beijing) and the assay was executed according to the manufacturer's instructions.

### 2.4 Serum lipid and transaminase measurements

Serum triglyceride (TG), total cholesterol (TCHO), low density lipoprotein cholesterol (LDL-C), high density lipoprotein cholesterol (HDL-C), ALT, aspartate aminotransferase (AST) measurements were performed using assay kits of A110–1, A111-1-1, A113-1-1, A112-1-1, C009-2, and C010-2 respectively from Nanjing Jiancheng Bioengineering Institute as per manufacturer instructions.

### 2.5 Network pharmacology

YCHT active chemical components were the mutual ingredients compiled from TCMSP^16^ (the oral bioavailability-OB ≥ 15% and the drug-likeness-DL ≥ 0.2), BATMAN^17^ (default set), and TCMID^18^ (default set). NAFLD and fat metabolism associated targets were acquired from GeneCards^19^ (score >10). YCHT related targets were screened from BATMAN (score >20) and TCMSP. All the targets were unified in UniProt.^20^ Target protein-protein interaction (PPI) networks were obtained from STRING.^21^ The compounds to targets networks and PPI networks were constructed by using Cytoscape software (V 3.7.2.). The target gene pathway enrichment analysis was accomplished by using the Metascape^22^ database, and the graph was created by the ggplot 2 package in R language (V 4.2.0).

### 2.6 RNA extraction and qRT-PCR analysis

Total liver RNA was extracted by RNAiso Plus (TaKaRa Biotechnology, Dalian) according to the manufacturer's protocol. Complementary DNA (cDNA) was synthesized from total RNA using Prime Script RT Master Mix (TaKaRa Biotechnology, Dalian). Real-time PCR for liver cDNA was performed on a 7500 Real-Time PCR System (Applied Biosystems, Foster City, USA) with SYBR Green premix EX TaqTM (TaKaRa Biotechnology, Dalian). The result for each specific gene was normalized to the ribosome 18s RNA gene. The 2^-ΔΔCt^ method^23^ was used to calculate the relative fold change of each gene. The primers used in this study were shown in Table 1.

**Table 1:** Primer sequences for qRT-PCR.

Gene	Forward Sequences (5′-3′)	Reverse Sequences (5′-3′)
RN18s	GACTCAACACGGGAAACCTCACC	ACCAGACAAATCGCTCCACCAAC
ADIPOQ	CCAATGTACCCATTCGCTTTAC	GAAGTAGTAGAGTCCCGGAATG
PPARG	TCCTGTAAAAGCCCGGAGTAT	GCTCTGGTAGGGGCAGTGA
IGF1	CTGGACCAGAGACCCTTTGC	GGACGGGGACTTCTGAGTCTT
PPARA	AGAGCCCCATCTGTCCTCTC	ACTGGTAGTCTGCAAAACCAAA
MTHFR	GGCAGCGAGAGTTCCAAGG	CAGGGAGAACCACTTGTCACC
SERPINE1	TTCAGCCCTTGCTTGCCTC	ACACTTTTACTCCGAAGTCGGT
NR1H4	GCAACCAGTCATGTACAGATTC	TTATTGAAAATCTCCGCCGAAC
APOA1	CTACCTTGAACGAGTACCACAC	CACTCTGGACTTGGGTCTTAAG
APOA2	AAGGAGCTTTGGTTAAGAGACA	AAGTTCACCAGACTAGTTCCTG

### 2.7 Western blot analysis

Liver protein samples were extracted by using appropriate RIPA lysis buffer containing 1 mM phenylmethylsulfonyl fluoride (PMSF) and phosphatase inhibitors (AR0102-100, Boster, Wuhan) based on the instructions of the manufacturer. The extracted protein concentrations were measured with a bicinchoninic acid (BCA) assay kit (AR0146, Boster, Wuhan). The protein samples were then mixed with SDS-PAGE loading buffer (AR1112, Boster, Wuhan) and boiled at 100 °C for 10 min. After being separated by 10% SDS-PAGE (AR0138-100, Boster, Wuhan), protein samples were transferred onto a PVDF membrane (Millipore, Billerica, USA). The membranes were blocked and then incubated individually with the primary antibodies of NR1H4 (bs-12867, Bioss, Beijing), APOA1 (bs-0849R, Bioss, Beijing), or beta-Actin (bsm-33036 M, Bioss, Beijing) at 4 °C overnight. After which, membranes were incubated with horseradish peroxidase (HRP) conjugated goat anti-rabbit IgG (BA1054, Boster, Wuhan) or HRP conjugated goat anti-mouse IgG (BA1050, Boster, Wuhan) secondary antibody for 2 h at room temperature. Enhanced chemiluminescence (ECL) (AR1197, Boster, Wuhan) was applied to visualize protein bands. Western blot images were captured on the FluorChem FC3 system (Protein Simple, San Jose, USA). Protein bands from 3 to 4 animals were analyzed manually by AlphaView software and normalized to beta Actin (ACTB) to ensure equal protein loading.

### 2.8 Immunohistochemistry assay

The liver paraffin sections were de-paraffinization in xylene and rehydration through graded ethanol, then washed with distilled water for 5 min and incubated with 3% hydrogen peroxide for 20 min to block endogenous peroxidase. Followed by 10 min by 3 times of PBS washing, antigen retrieval was performed to the sections. Next, sections were washed with PBS for 10 min by 3 times and blocked with 5% bovine serum albumin (BSA) for 30 min at room temperature. Samples were incubated with polyclonal rabbit anti-NR1H4 primary antibody (1:200, bs-12867, Bioss, Beijing) or polyclonal rabbit anti-APOA1 primary antibody (1:200, bs-0849R, Bioss, Beijing) at 4 °C overnight. After 10 min by 3 times PBS washing, the labeled avidin-biotinylated rabbit IgG kit (SA1022, Boster, Wuhan) was used for amplification of primary antibody binding. The antibody-antigen complex was visualized via diaminobenzidine (DAB) (AR1022, Boster, Wuhan) for 5 min at room temperature. Sections were counterstained with Harris hematoxylin, then dehydrated through graded ethanol, and finally sealed with neutral balsam.

### 2.9 Images analysis and statistics

The images of liver oil red O staining, PAS staining, and immunohistochemical staining were captured by an Olympus BX41 (Tokyo, Japan) microscope. Liver lipid level and glycogen expression level were evaluated semi-quantitatively by Image J software (V 1.8.0) based on the positive staining area/total captured area.

The sections used for oil red O and PAS analyses were randomly picked from 3 to 5 animals of each group, and the picture number used for each analysis was marked as per figure legend.

Data were reported as the mean ± SEM. The statistical data were processed by GraphPad Prism 8 software. The *t*-test was used for comparison between two groups, and the variance between multiple groups was analyzed by using one-way ANOVA followed by Tukey's post-hoc test. P < 0.05 was considered as significant difference.

## 3 Results

### 3.1 YCHT decreased the body weight and liver weight of high fat diet induced NAFLD mice

**Fig. 1:**
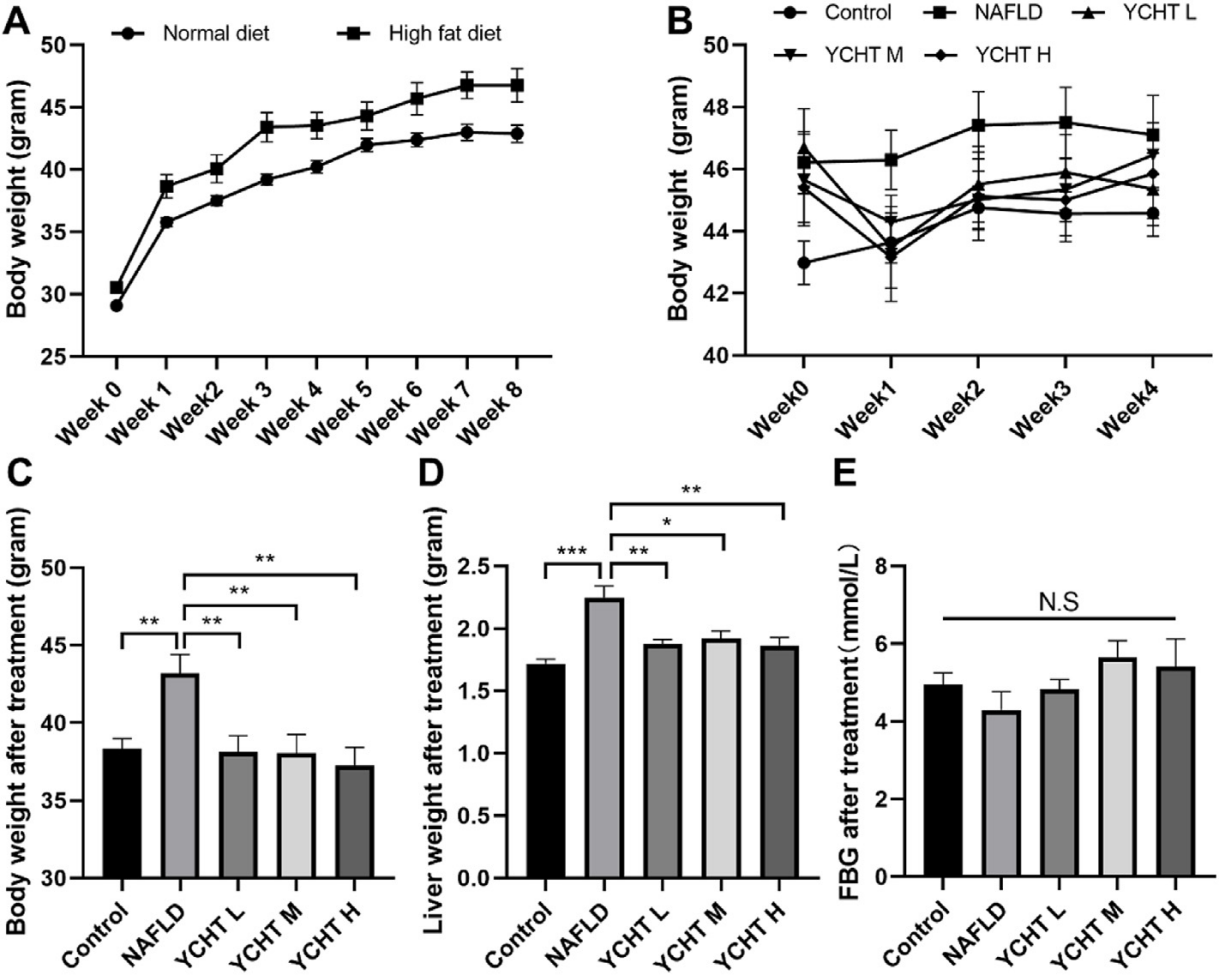
YCHT decreased body weight and liver weight of HFD induced NAFLD mice. (A) Body weight during HFD treatment. (B) Body weight during YCHT treatment. (C) Body weight after YCHT treatment. (D) Liver weight after YCHT treatment. (E) Average FBG level after YCHT treatment. ∗p < 0.05, ∗∗p < 0.01, ∗∗∗p < 0.001; n = 7–10.

The body weight of both control and NAFLD mice increased gradually during the first 8 weeks, while NAFLD mice became heavier than controls by the second week (Fig. 1A). After YCHT treatment, the body weight of NAFLD mice started to drop (Fig. 1 B) and at the end of the treatment, both body weight and liver weight in YCHT treated groups were significantly decreased compared to the NAFLD group (Fig. 1C–D). However, the mice's FBG level was not affected by either HFD or YCHT treatment in this study (Fig. 1E).

### 3.2 YCHT suppressed liver lipid accumulation of NAFLD mice

**Fig. 2:**
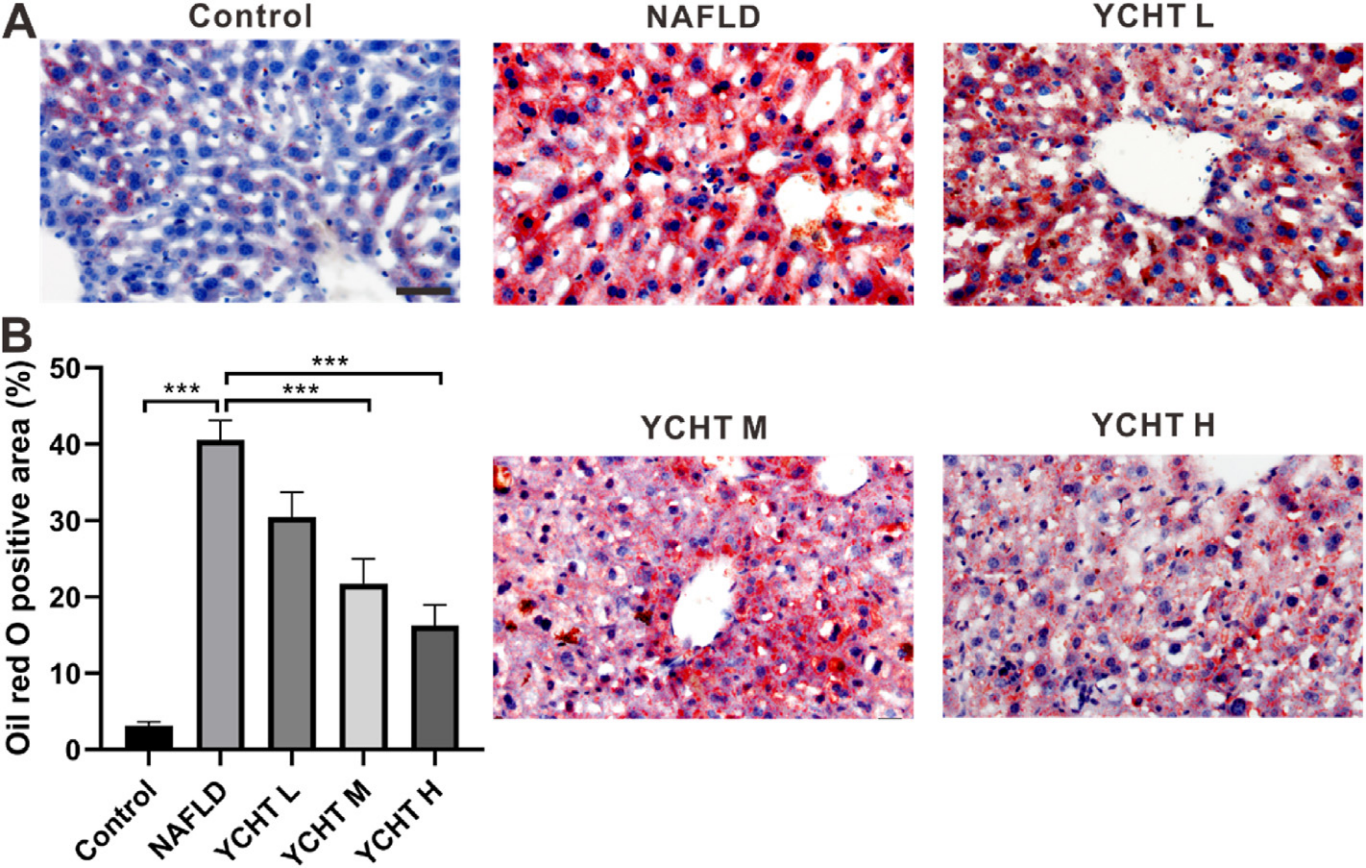
YCHT decreased the liver lipid level of NAFLD mice. (A) Oil red O staining. (B) Semi-quantitative analysis of liver lipid level. ∗∗∗p < 0.001; scale bar: 50 μm; image at 400 × ; n = 5.

The oil red O positive area represents the actual lipid stored in liver tissue, so oil red O staining was proposed as the standard method for quantifying steatosis in NAFLD liver.^24^ To evaluate the hepatic lipid level changes, oil red O staining was performed. In the control group, oil red O positive staining only presented in limited numbers of hepatocytes; the NAFLD liver was pervasively stained with oil red O in dark red color; oil red O positive staining in YCHT L liver was in the form of droplets, and universally scattered in cell somas; the dark stained oil red O positive area was mainly distributed around the central vein or portal triad in YCHT M liver; in YCHT H, oil red O staining sparsely situated in some hepatocytes as small droplets (Fig. 2A). In comparison with the control, the liver lipid level of NAFLD group was increased from 3% to about 40%; YCHT in middle or high concentration can significantly decrease the oil red O positive area of NAFLD, indicating the reduction of lipid storage in the liver (Fig. 2B).

### 3.3 YCHT increased liver glycogen level and improved liver pathology of NAFLD mice

**Fig. 3:**
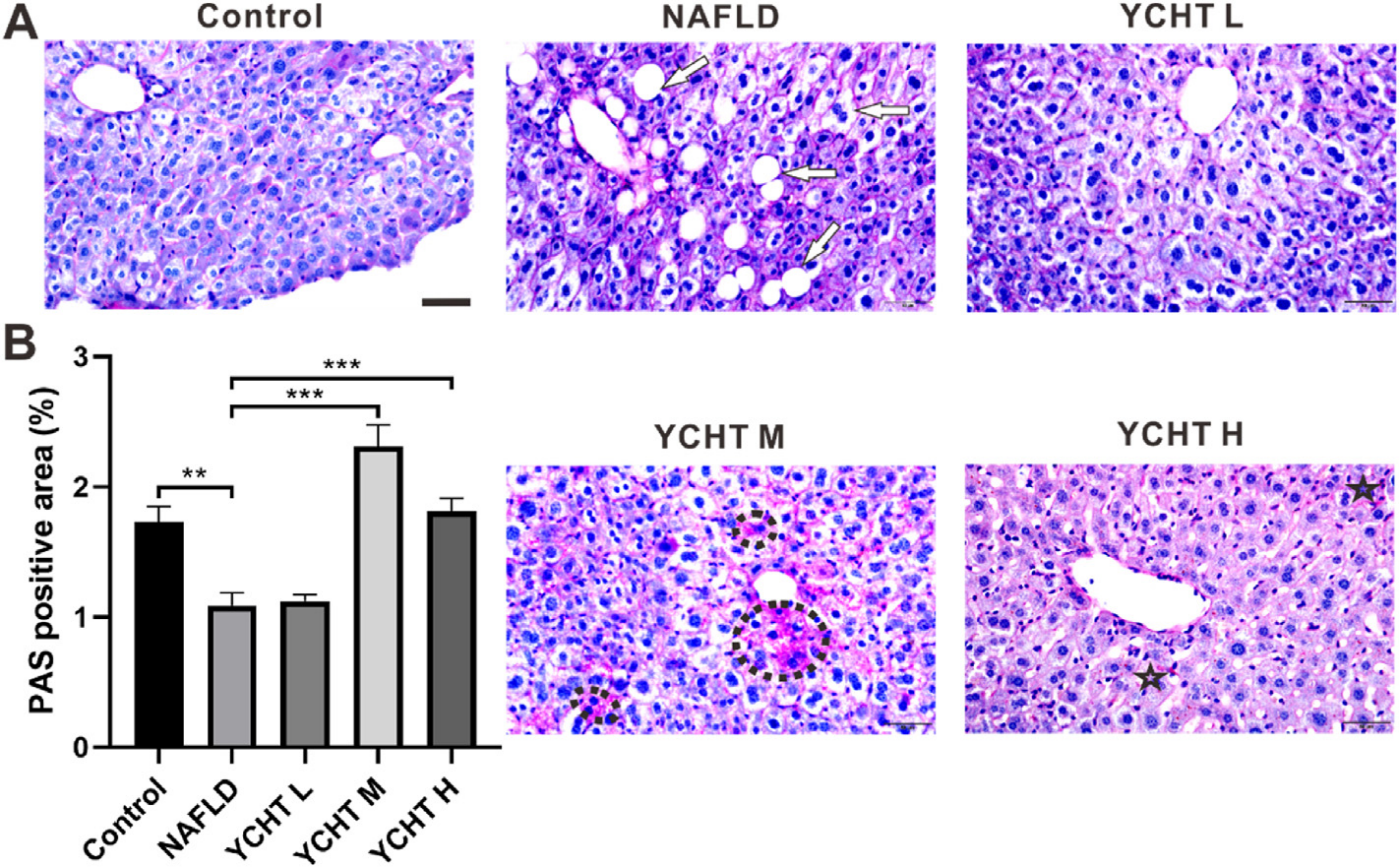
YCHT increased the liver glycogen level of NAFLD mice. (A) Liver glycogen as demonstrated by PAS staining. (B) Semi-quantitative analysis of liver glycogen level. ∗∗∗p < 0.001; scale bar: 50 μm; image at 400 × ; n = 25.

To investigate the liver glycogen changes after YCHT treatment, PAS staining was performed. In the control group, liver glycogen was stained as dark pink color and located in the cytoplasm; PAS positive staining was recognized in limited cell plasma of the NAFLD group, while the steatosis and cell ballooning were detected all over the liver (Fig. 3A white arrow); some hepatocytes in the YCHT L group were positively stained, and a few small fat vacuoles were also observed; glycogen was stained as a dark red color in liver soma of the YCHT M group, (Fig. 3A black circle), and fat vacuole was barely recognized; glycogen in YCHT H group was universally dispersed in the cytoplasm (Fig. 3A black star). There was about 1.7% of glycogen in the liver of the control group, NAFLD caused glycogen reduction can be restored by middle and high dose YCHT treatment but not by low dose YCHT (Fig. 3B).

### 3.4 YCHT ameliorated serum lipid disorder of NAFLD mice

**Fig. 4:**
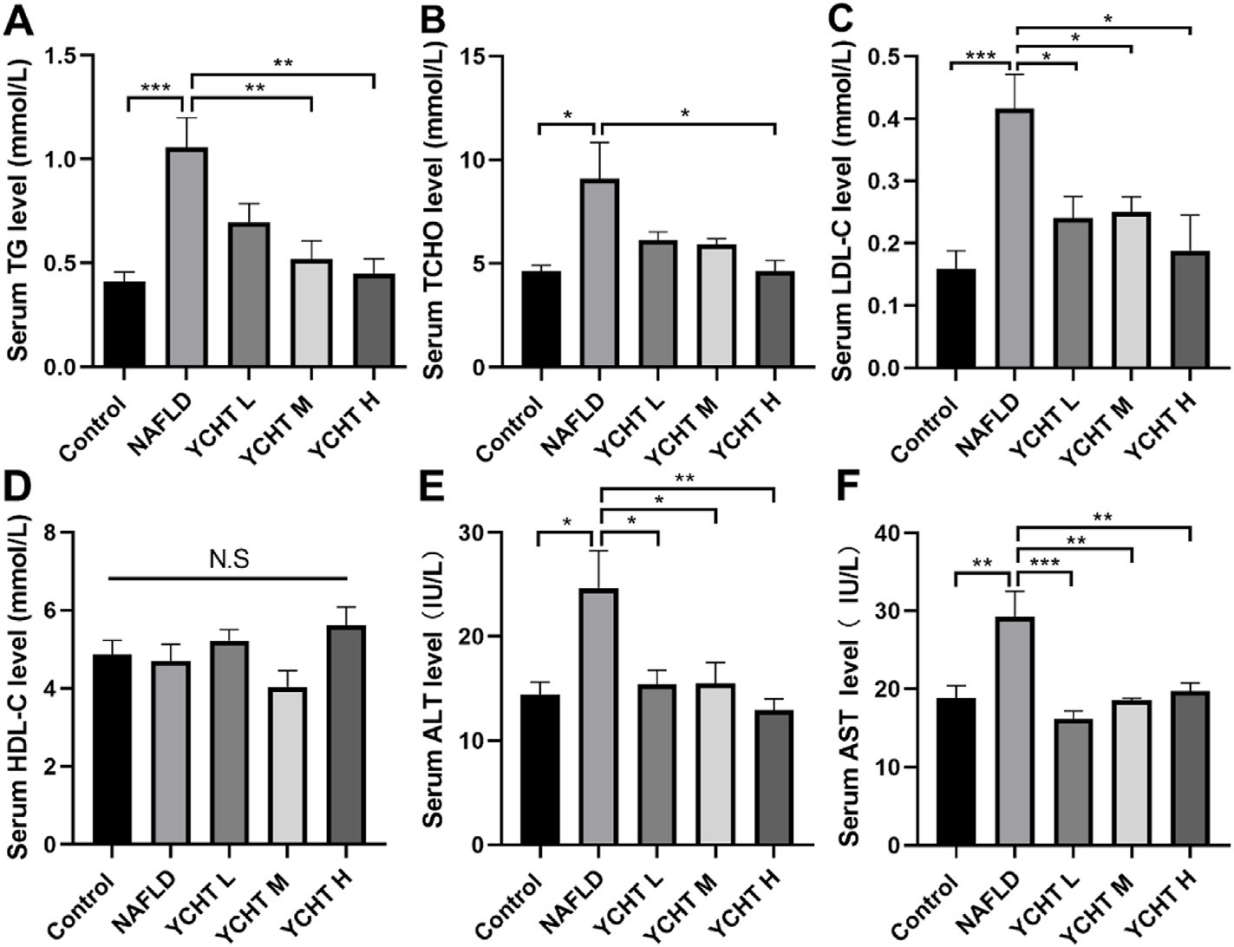
YCHT ameliorated serum lipid disorder of NAFLD mice. (A) YCHT decreased NAFLD serum TG level. (B) YCHT H decreased NAFLD serum total cholesterol level. (C) YCHT decreased NAFLD serum LDL-C level. (D) Serum HDL-C did not change after NAFLD or YCHT treatment. (E) YCHT decreased NAFLD serum AST level. (F) YCHT decreased NAFLD serum ALT level. ∗p < 0.05, ∗∗p < 0.01, ∗∗∗p < 0.001; n = 4–8.

Serum lipid level is a key indicator for NAFLD occurrence and progress. To determine the changes in serum lipid levels, serum TG, TCHO, LDL-C, and HDL-C were measured. Serum ALT and AST levels which commonly reflect the progress of NAFLD were also tested. When compared to the control group, NAFLD dramatically elevated serum TG level, whereas YCHT treatment reduced HFD increased serum TG level (Fig. 4A). NAFLD notably boosted the serum TCHO level of control mice, and YCHT diminished the raised serum TCHO level but only YCHT H was statistically different from the NAFLD group (Fig. 4B). The LDL-C level in the NAFLD group was markedly higher than in the control group and YCHT efficiently down-regulated LDL-C levels (Fig. 4C). HDL-C levels in control mice did not substantially change after HFD or YCHT treatment. (Fig. 4D). Both serum ALT and AST levels were elevated in the NAFLD group when compared to the control, YCHT in all the 3 concentrations suppressed the ALT and AST levels that were increased by NAFLD (Fig. 4E and F).

### 3.5 Network-based pharmacological analysis of YCHT ameliorated HFD induced NAFLD

The active ingredients of YCHT variant from different databases. The mutual active ingredients screened from multiple Traditional Chinese medicines (TCM) chemical databases stand for the stable substances contained in YCHT. After comparing the YCHT ingredients contained in TPMSP, TCMCID, and BATMAN, 25 mutual compounds were chosen as the active ingredients of YCHT (Table 2).

**Table 2:** Active compounds of YCHT.

NO.	Active ingredients	Herb name	OB	DL
1	Beta-Sitosterol	YINCHEN	36.91	0.75
2	Capillarisin	YINCHEN	57.56	0.31
3	Eupatolitin	YINCHEN	42.55	0.37
4	Genkwanin	YINCHEN	37.13	0.24
5	Isorhamnetin	YINCHEN	49.6	0.31
6	Quercetin	YINCHEN	46.43	0.28
7	Crocetin	ZHIZI	35.3	0.26
8	Geniposide	ZHIZI	14.64	0.44
9	Ursolic acid	ZHIZI	16.77	0.75
10	2-Cinnamoyl-Glucose	DAHUANG	17.02	0.22
11	3-Hydroxy-25-Norfriedel-3,1(10)-Dien-2-One-30-Oic Acid	DAHUANG	18.4	0.78
12	Aloe-Emodin	DAHUANG	83.38	0.24
13	Anthraglycoside B	DAHUANG	27.06	0.8
14	Chrysophanol Glucoside	DAHUANG	20.06	0.76
15	Emodin	DAHUANG	24.4	0.24
16	Emodin-6-Glucoside	DAHUANG	16.09	0.8
17	Gallic Acid-4-O-(6′-O-Galloyl)-Glucoside	DAHUANG	27.06	0.67
18	Mutatochrome	DAHUANG	48.64	0.61
19	Palmidin A	DAHUANG	32.45	0.65
20	Physcion	DAHUANG	22.29	0.27
21	Procyanidin B-5,3′-O-Gallate	DAHUANG	31.99	0.32
22	Rhein	DAHUANG	47.07	0.28
23	Rheinoside A	DAHUANG	26.28	0.75
24	Sennoside E	DAHUANG	50.69	0.61
25	Toralactone	DAHUANG	46.46	0.24

**Fig. 5:**
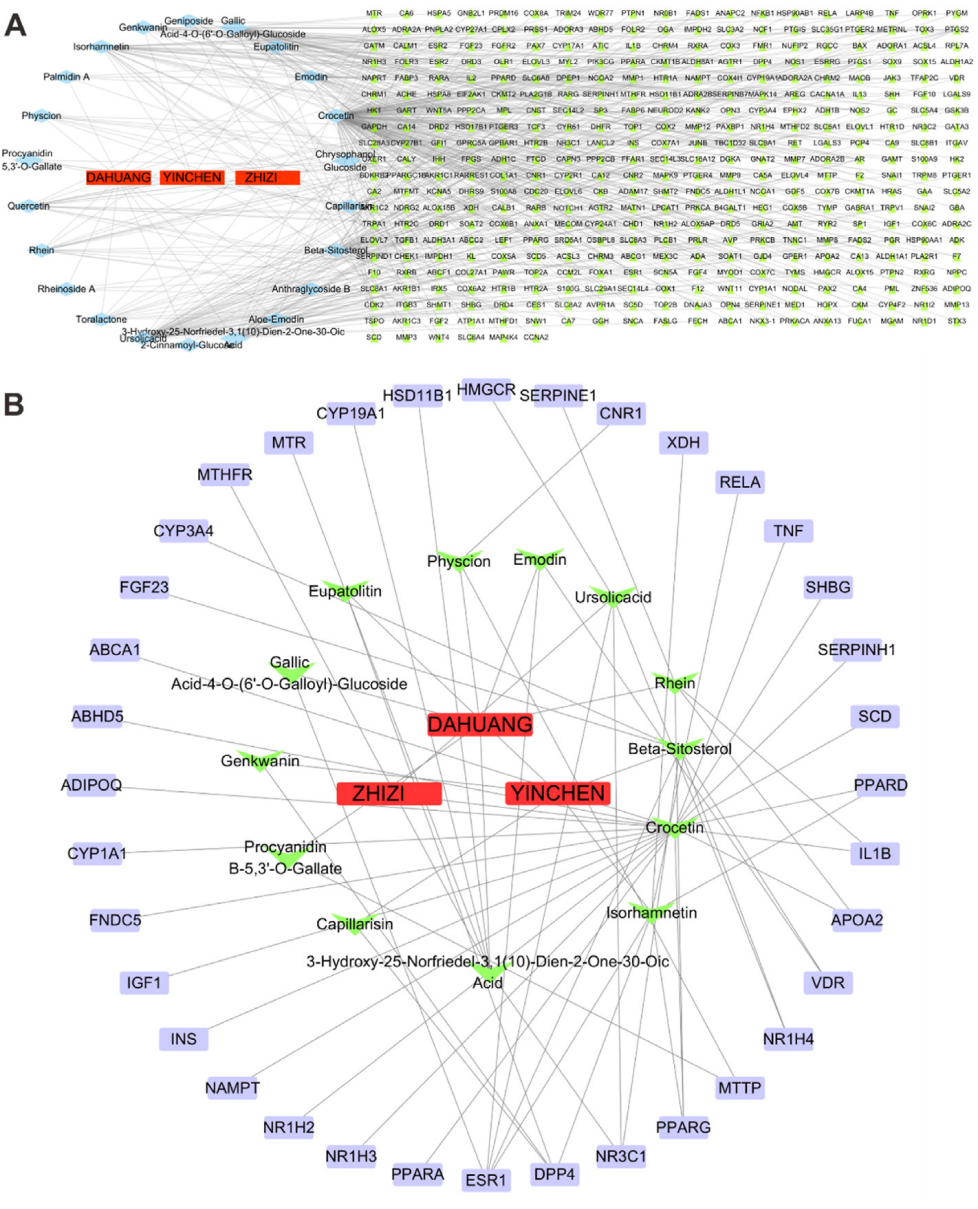
Screening the potential targets of YCHT for NAFLD modulation. (A) Network of YCHT active compounds to targets. (B) Network of YCHT active compounds to NAFLD and fat metabolism targets.

From the screened 25 active compounds of YCHT, 22 compounds are linked to 489 potential targets (Fig. 5A). Based on the results that YCHT improved liver steatosis and serum lipid level, NAFLD and fat metabolism associated targets were collected from the GeneCards database. When comparing 489 YCHT related targets to the candidate targets of NAFLD and fat metabolism, the overlapped 35 targets which refer to 13 active compounds are potential goals for YCHT to modulate NAFLD (Fig. 5B).

**Fig. 6:**
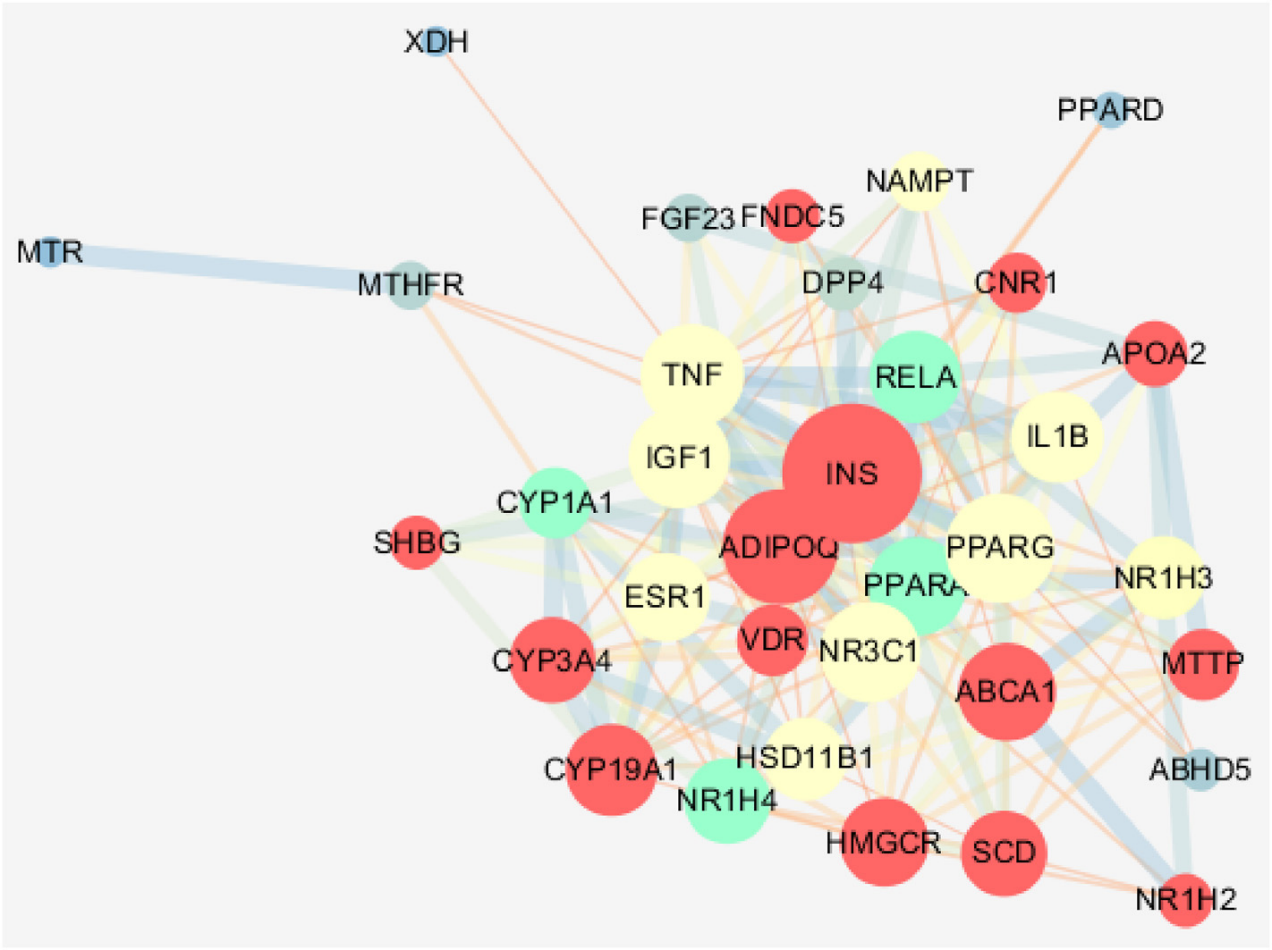
YCHT targets interaction for NAFLD modulation. The nodes represent targets and the edges represent the interactions between targets, node size is proportional to their degree. Different clusters are shown with different colors.

The 35 potential targets of YCHT for NAFLD and fat metabolism modulation were first analyzed by STRING to get PPI. The target PPI was further constructed by Cytoscape. Cytoscape MOCOD analysis revealed that the goal cluster consists of CYP1A, NR1H4, PPARA, and RELA which are involved in NAFLD amelioration (Fig. 6).

**Fig. 7:**
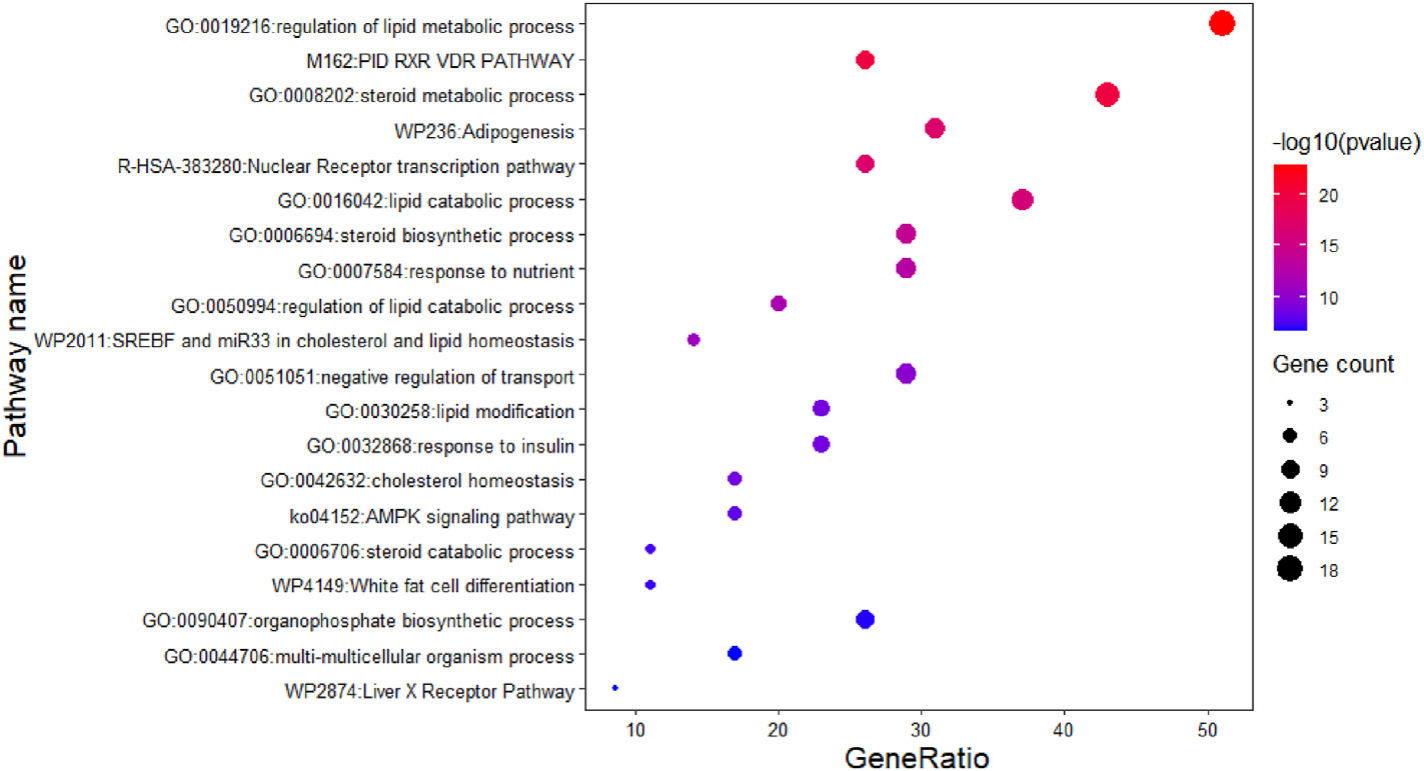
Metascape analysis for targets enrichment of biological pathway and process.

To understand the biological pathways and processes of the YCHT targets, the enrichment for 35 screened targets was analyzed by the Matascape database. The targets mainly involved in lipid metabolic regulation, RXR and VDR pathway, steroid metabolic process, and the top 20 processes are presented as a bubble graph in Fig. 7. The -log10 value (P-value) provided an enrichment score which indicated the importance of the gene pathway and process enrichment.

### 3.6 YCHT enhanced the NAFLD suppressed NR1H4 and APOA1 mRNA expression

**Fig. 8:**
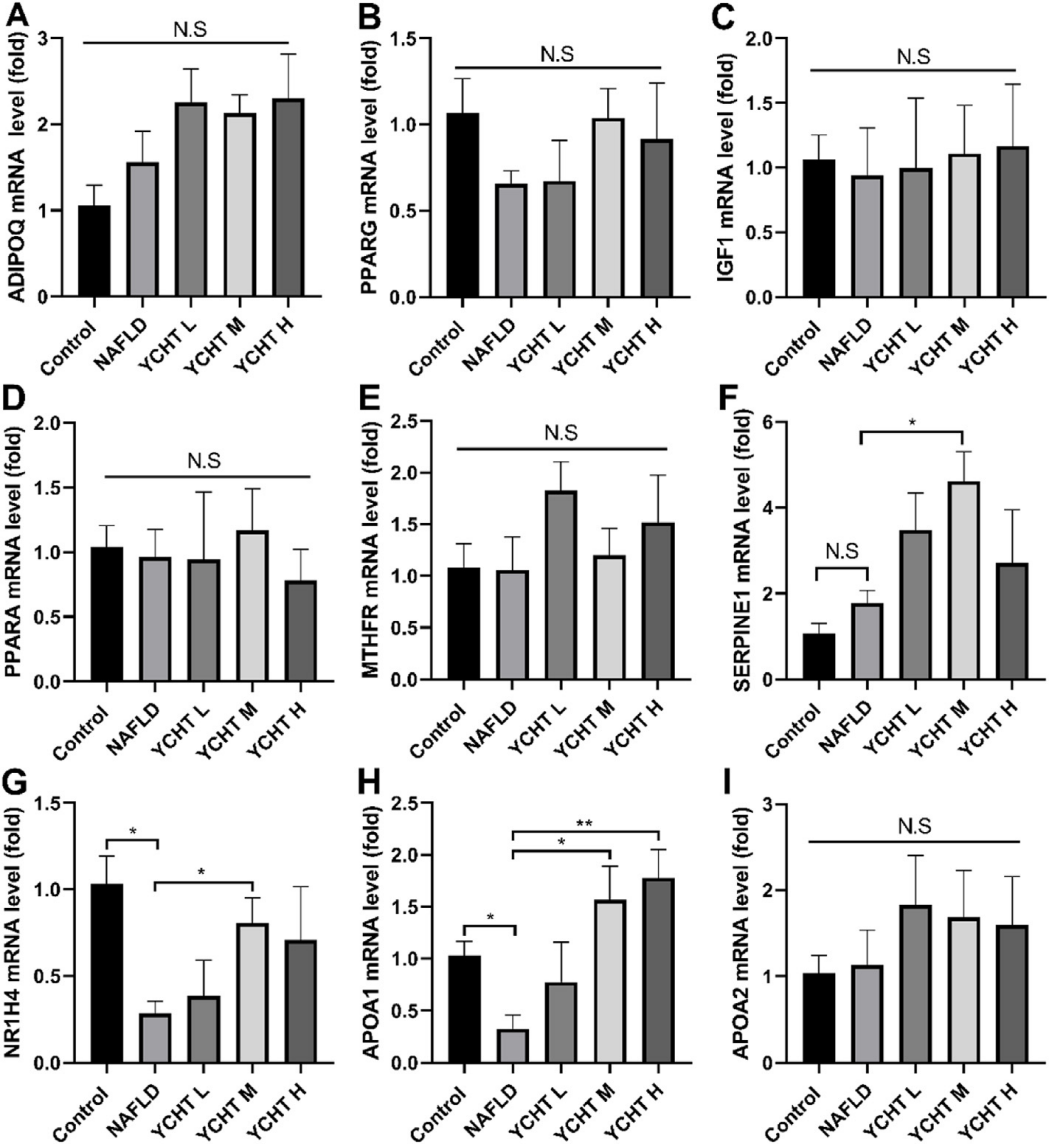
YCHT increased the NR1H4 and APOA1 gene expression. (A) Relative mRNA expression of ADIPOQ. (B) Relative mRNA expression of PPARG. (C) Relative mRNA expression of IGF1. D, Relative mRNA expression of PPARA. (E) Relative mRNA expression of MTHFR. (F) Relative mRNA expression of SERPINE1. (G) Relative mRNA expression of NR1H4. (H) Relative mRNA expression of APOA1. (I) Relative mRNA expression of APOA2. ∗p < 0.05, ∗∗p < 0.01; n = 3–4.

The network pharmacology analyses determined that there were 35 potential targets for YCHT to modulate NAFLD. To verify the gene expression pattern of lipid metabolism associated targets that also contribute to NAFLD regulation, ADIPOQ, PPARG, IGF1, PPARA, MTHFR, SERPINE1, NR1H4, and APOA2 mRNA levels were tested. Since YCHT has been proven to influence the lipid metabolism related protein APOA1,^25^ the APOA1 gene expression level was also tested. The mRNA level of ADIPOQ, PPARG, IGF1, PPARA, and MTHFR were not substantially altered by HFD or YCHT treatment (Fig. 8A–E). Compared to NAFLD the SERPINE1 mRNA expression was increased in the YCHT M group, but significance was not observed when making comparisons between other groups (Fig. 8F). NAFLD significantly reduced the gene expression of NR1H4 compared to the control, YCHT treatment could raise the NR1H4 mRNA level, but only YCHT M was statistically different from the NAFLD group (Fig. 8G). The APOA1 mRNA level in the NAFLD group was only about 0.32-fold as the control, YCHT in middle and high doses markedly promoted the APOA1 gene expression of NAFLD mice (Fig. 8H). The APOA2 mRNA level was not affected by HFD, and YCHT treatment did not essentially increase the APOA2 expression (Fig. 8I).

### 3.7 YCHT compensated the NAFLD suppressed NR1H4 and APOA1 protein level

**Fig. 9:**
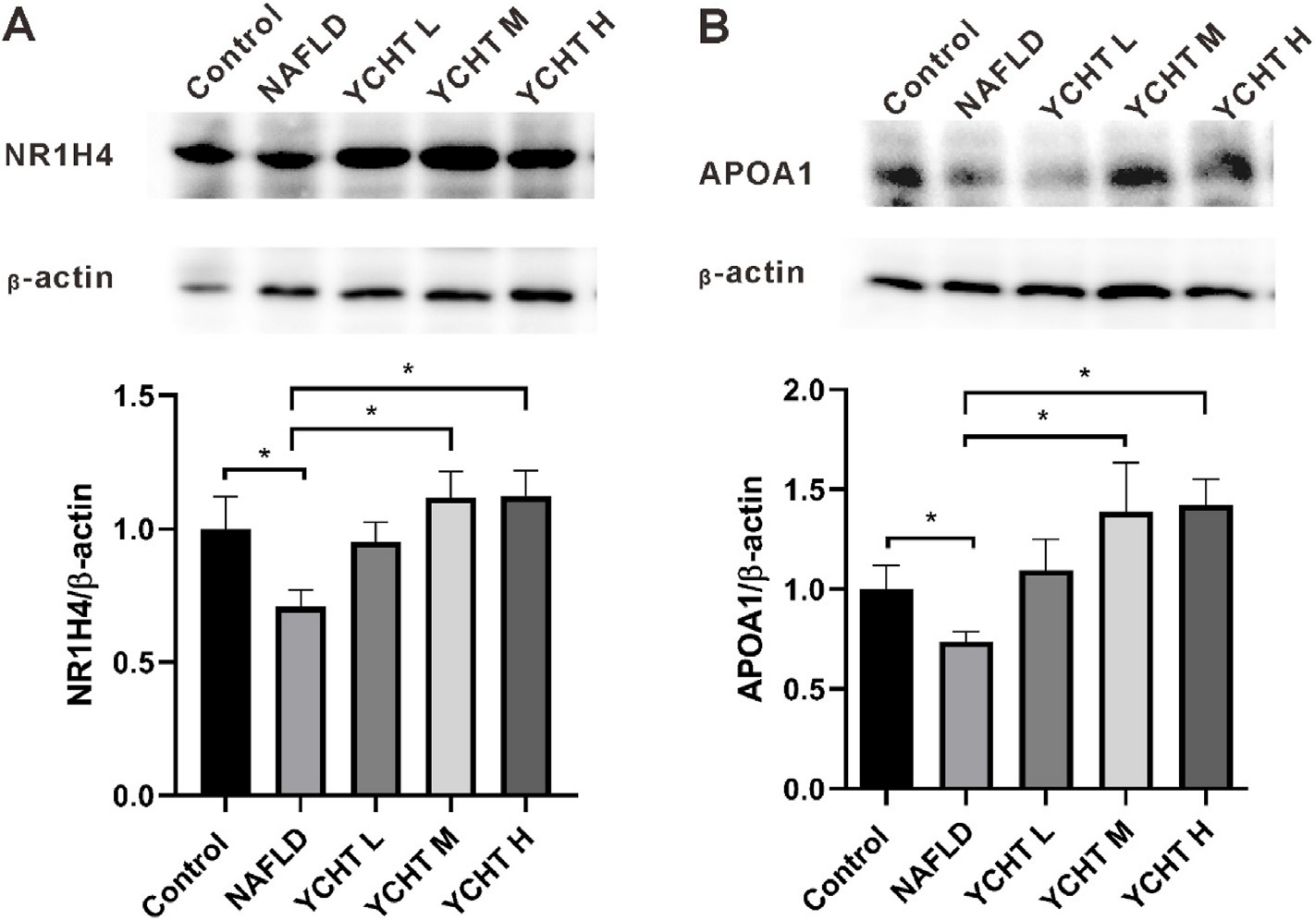
YCHT restored NR1H4 and APOA1 expression of NAFLD. (A) NR1H4 protein expression pattern. (B) APOA1 protein expression pattern. ∗p < 0.05; n = 3–4.

YCHT treatment elevated the NAFLD suppressed NR1H4 and APOA1 gene expression. In order to identify changes of NR1H4 and APOA1 in protein level, Western blot was performed. Compared to the control, both NR1H4 and APOA1 expression were down-regulated in the NAFLD group, YCHT M and YCHT H treatment significantly increased the NR1H4 and APOA1 protein levels of NAFLD mice (Fig. 9A–B).

### 3.8 YCHT recovered the localization pattern of NR1H4 and APOA1 in the liver

**Fig. 10:**
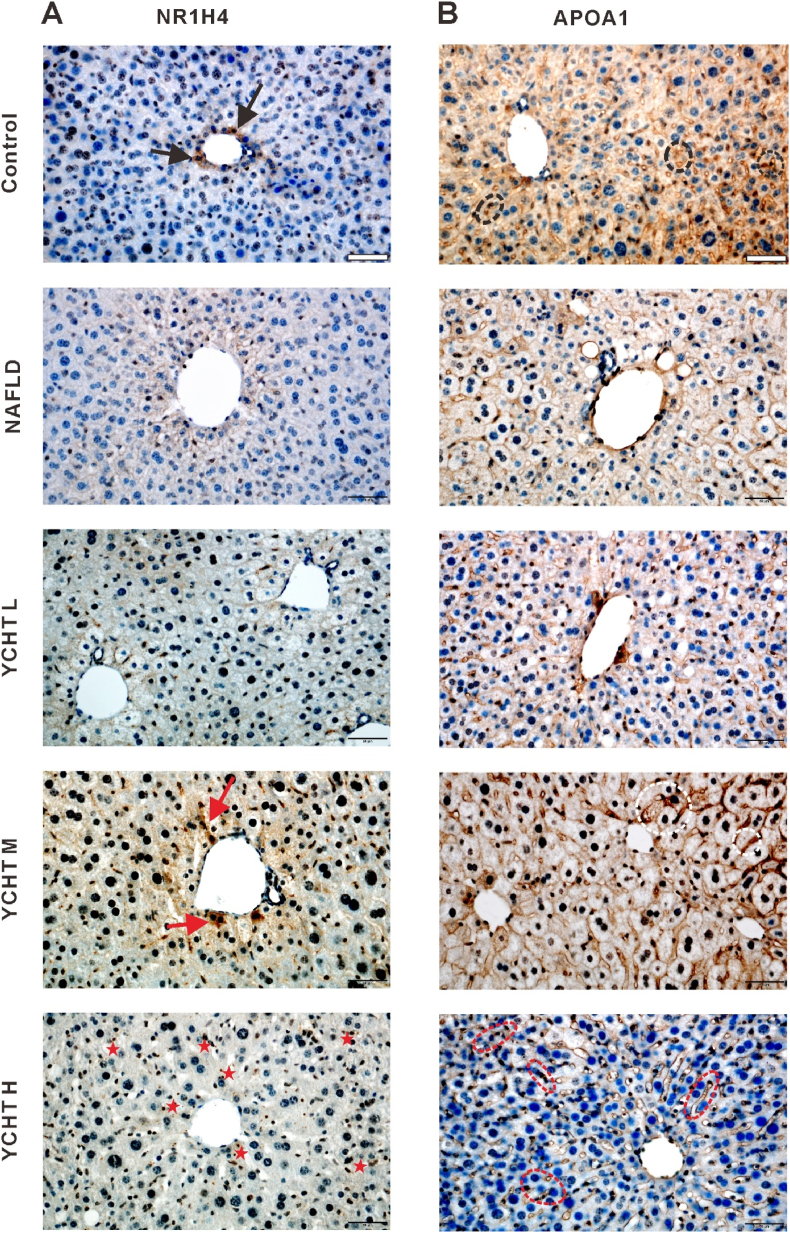
NR1H4 and APOA1 localization pattern in the liver. (A) NR1H4 expression pattern in the liver. (B) APOA1 expression pattern in the liver. Scale bar: 50 μm; image at 400 × .

To visualize the in-suit expression pattern of NR1H4 and APOA1 in the liver, IHC staining was performed. In the control group, NR1H4 was mainly expressed in the nuclei of hepatocytes, at the same time NR1H4 positive signals were frequently noticed in the cytoplasm of some hepatocytes around the central veins (Fig. 10A, black arrow). In the NAFLD group, NR1H4 positive signals presented in the nuclei of limited hepatocytes around the central veins. NR1H4 in YCHT L was dark brown stained and also found in the hepatocyte nuclei. The positive staining of NR1H4 in YCHT M was not only strongly expressed in cell nuclei but also distributed in some cytoplasm (Fig. 10A, red arrow). In the YCHT H, NR1H4 was dyed dark brown color and restricted in the cell nucleus (Fig. 10A, red star).

The APOA1 in the control group was stained with dark brown color and universally dispersed in liver sinusoids and cell plasma (Fig. 10B, black dotted circle). In the NAFLD group, APOA1 was dyed light brown and primarily identified in hepatic sinusoids (Fig. 10B). APOA1 in YCHT L was not only expressed in liver sinusoids but also the hepatocytes around the central vein (Fig. 10B). In the YCHT M group, APOA1 was colored in dark brown color and sited in cell plasma, extracellular matrix, and liver sinusoids (Fig. 10B, white dotted circle). The positive APOA1 signal from YCHT H was mainly detected in hepatic sinusoids (Fig. 10B, red dotted circle).

## 4 Discussion

As a result of the high calorie food intake and lack of exercise, NAFLD patients were increasing dramatically in recent years. However, there are limited medications available for NAFLD treatment. TCM exhibited great potential in treating NAFLD as alternative medicine. A newly released study indicated that about 20%–30% of patients are using herbal medicine to treat chronic liver diseases worldwide.^26^ YCHT, a famous Chinese medicine formula displayed protective properties in liver diseases such as NAFLD, hepatocyte apoptosis,^27^ and liver fibrosis.^14^^,^^28^

Previous studies normally used single dose YCHT to treat NAFLD and diverse dosages were chosen.^8^^,^^11^ Different groups used different YCHT concentrations and displayed different results, therefore it is not clear whether that was due to dose effects. To determine the YCHT dose effect for NAFLD, 3 different YCHT concentrations were prepared. Kunming mice were selected to generate the NAFLD model, considering that Kunming mice could efficiently accumulate body weight and liver adipose after HFD feeding.^29^

YCHT of all the 3 concentrations can significantly suppress the HFD caused body weight and liver weight elevation (Fig. 1). The results from oil red O staining indicated that YCHT ameliorated NAFLD induced liver steatosis and significantly diminished liver lipid storage (Fig. 2). These results were consistent with the studies from others research that YCHT can improve hepatic triglyceride metabolism^9^ and significantly reduced hepatic steatosis and ballooning degeneration.^10^ Oil red O staining was chosen to detect liver steatosis patterns and triglyceride levels because it is more sensitive and accurate in determining steatosis compared to HE staining.^30^ Further study in PAS staining revealed that as the NAFLD induced steatosis was decreased in the liver, the glycogen level in YCHT M and YCHT H groups were significantly increased (Fig. 3), which implies that YCHT suppressed liver lipid accumulation by promoting lipid to glycogen transformation.

Serum lipid level is a very important feature to reflect the progression of NAFLD. In general, serum lipid level was greatly increased in NAFLD mice and YCHT can improve NAFLD caused lipid metabolism disorder. YCHT in all the 3 concentrations reduced serum TG levels, although only YCHT M or YCHT H was statistically different from the NAFLD (Fig. 4A). Nevertheless, only YCHT H can significantly reduce the TCHO level of NAFLD (Fig. 4B). Similar results were reported that YCHT reduced serum TG levels but did not extensively decrease the TCHO levels of NAFLD rats.^8^ In addition, YCHT also reduced the raised serum ALT and AST levels in NAFLD (Fig. 4E and F) which exhibited its potential to suppress NAFLD progression and ameliorate liver damage.

In this study, reverse network pharmacology was applied to dig out the candidate targets for YCHT to modulate NAFLD. Contrasting with the active compounds to pathway strategy of the traditional network pharmacology,^31^ the experimental evidence of YCHT on HFD induced NAFLD was first investigated. Based on the beneficial results that YCHT decreased serum lipid levels and improved liver steatosis, fat metabolism and NAFLD related targets were gathered. After comparing the collected targets with YCHT related targets, the mutual targets were elected as the goal targets of YCHT for NAFLD modulation. YCHT had been shown to affect rat APOA1 protein which participated in lipid metabolism.^25^ Thus, APOA1 was also included as the YCHT potential target for NAFLD modulation. The reason why APOA1 has not comprised the YCHT targets could be because different TCM database contains different active components or the settings in TCM databases rule out some active compounds which could be related to APOA1. For the screened targets, NAFLD suppressed NR1H4 and APOA1 expression was upregulated by YCHT treatment in both gene and protein levels (Fig. 8 G, H; Fig. 9). So that NR1H4 and APOA1 were the experimentally validated targets for YCHT to modulate HFD induced NAFLD.

NR1H4 (Farnesoid X receptor, FXR) is the bile acid-activated nuclear receptor that regulates glucose and lipid homeostasis. When lacking NR1H4, mice developed severe fatty liver and elevated circulating FFAs.^32^ This research disclosed that HFD suppressed NR1H4 expression and YCHT could relieve NAFLD by rescuing the NR1H4 expression. The re-activation of NR1H4 represses bile acid synthesis.^33^ To compensate for NR1H4 induced bile acid deficiency, the liver accelerates the conversion of cholesterol to bile acids. The process of cholesterol to bile acids transformation consumes the circulating cholesterol which leads to a drop in blood cholesterol level.^34^ On the other hand, NR1H4 also closely links lipid metabolism to glucose metabolism. NR1H4 knockout mice manifested high serum TG and FFA levels, in association with impaired glucose tolerance and insulin resistance.^35^ Furthermore, NR1H4 agonists upregulated lipid metabolism and increased glycogen synthesis.^36^ In this study, YCHT also demonstrates the ability to enhance hepatic glycogen (Fig. 3B) which could be the downstream effects of the NR1H4 upgrade.

Our results manifested that YCHT at middle and high doses can compensate for the APOA1 expression suppressed by NAFLD. The APOA1 level was negatively associated with the total cholesterol and LDL-C level (Fig. 4C and D). APOA1 triggers lecithin-cholesterol acyltransferase (LCAT),^37^ which promotes cholesterol to be transported and excreted from the liver. APOA1 also mediates the cellular synthesis of HDL to increase the capacity of HDL, so that it can carry and potentially eliminate cholesterol from tissues,^38^ as a result, decreasing the serum LDL-C level. Taking together, YCHT restrains NAFLD progression via upregulating the expression of NR1H4 and APOA1 to modulate lipid metabolism mainly through the cholesterol pathway.

## 5 Conclusions

In conclusion, YCHT can significantly reduce body weight and improve the lipid metabolism of HFD induced NAFLD. Reverse network pharmacology screened out NR1H4 and APOA1 as the potential targets for YCHT to inhibit NAFLD. YCHT ameliorates NAFLD by boosting the expression of NR1H4 and APOA1 in both RNA and protein levels. YCHT can effectively prevent HFD induced NAFLD by modulating the promising targets of NR1H4 and APOA1.

## Authors’ contributions

Li Xu designed and conducted the experiments, and article writing; Hongliang Cui treated animals and performed IHC experiments. All authors approved the final manuscript.

## Funding

This work was supported by the grant from Scientific Research Plan Projects of the education department of Shaanxi province (19JK0243), P. R. China; National College Students' innovation and entrepreneurship training program (S202010716012).

## Declaration of competing interest

The authors declare that they have no conflicts of interest.
